# MicroRNA and Pathogenesis of Enterovirus Infection

**DOI:** 10.3390/v8010011

**Published:** 2016-01-06

**Authors:** Bing-Ching Ho, Pan-Chyr Yang, Sung-Liang Yu

**Affiliations:** 1 Department of Clinical Laboratory Sciences and Medical Biotechnology, College of Medicine, National Taiwan University, No. 1 Chang-Te Street, Taipei 10048, Taiwan; f94424002@ntu.edu.tw; 2 Center of Genomic Medicine, National Taiwan University, Taipei 10048, Taiwan; pcyang@ntu.edu.tw; 3 Department of Internal Medicine, National Taiwan University Hospital, Taipei 10048, Taiwan; 4 Institute of Biomedical Sciences, Academia Sinica, Taipei 10048, Taiwan; 5 Center for Optoelectronic Biomedicine, College of Medicine, National Taiwan University, Taipei 10048, Taiwan; 6 Graduate Institute of Pathology, College of Medicine, National Taiwan University, Taipei 10048, Taiwan; 7 Department of Laboratory Medicine, National Taiwan University Hospital, Taipei 10048, Taiwan

**Keywords:** non-coding RNA, microRNA, apoptosis, protein synthesis shutdown, virus replication

## Abstract

There are no currently available specific antiviral therapies for non-polio Enterovirus infections. Although several vaccines have entered clinical trials, the efficacy requires further evaluation, particularly for cross-strain protective activity. Curing patients with viral infections is a public health problem due to antigen alterations and drug resistance caused by the high genomic mutation rate. To conquer these limits in the development of anti-Enterovirus treatments, a comprehensive understanding of the interactions between Enterovirus and host cells is urgently needed. MicroRNA (miRNA) constitutes the biggest family of gene regulators in mammalian cells and regulates almost a half of all human genes. The roles of miRNAs in Enterovirus pathogenesis have recently begun to be noted. In this review, we shed light on recent advances in the understanding of Enterovirus infection-modulated miRNAs. The impacts of altered host miRNAs on cellular processes, including immune escape, apoptosis, signal transduction, shutdown of host protein synthesis and viral replication, are discussed. Finally, miRNA-based medication provides a promising strategy for the development of antiviral therapy.

## 1. Introduction

Enterovirus (EV) infections have emerged as a major public health problem, and EV outbreaks occur frequently in summer and early fall as epidemics throughout the world [1,2,3,4,5,6,7,8,9,10,11,12,13]. Human EVs are divided into seven species: Enterovirus species A (Coxsackie A viruses 2–8, 10, 12, 14, and 16 and Enteroviruses 71, 76, 89–92, 114, and 119), Enterovirus species B (Coxsackie A virus 9; Coxsackie B viruses 1–6; Echoviruses 1–7, 9, 11–21, 24–27, and 29–33; and Enteroviruses 69, 73–75, and 77–88), Enterovirus species C (Polioviruses 1–3 and Coxsackie A viruses 1, 11, 13, 15, 17–22, and 24), Enterovirus species D (Enteroviruses 68 and 70), Rhinovirus species A (Rhinovirus A viruses 1, 2, 7–13, 15, 16, 18–25, 28–34, and 36), Rhinovirus species B (Rhinovirus B viruses 3–6, 1, 17, 26, and 27) and Rhinovirus species C (Rhinovirus C viruses 1–51). These viruses depend on the host’s physiological features, including age, sex, immune response and nutritional status [14,15]. EVs can infect people of all age groups, but they severely affect younger children in particular and occasionally cause permanent paralysis and neurological complications [16]. Growing evidence indicates that EVs have caused symptomatic infections in North America, Malaysia, Singapore, Australia, and Taiwan [11,17,18,19,20]. Enterovirus 71 (EV71), a member of Enterovirus species A, is a non-enveloped virion with positive-strand RNA. EV71 was first identified in California in 1969 and has caused several outbreaks in the United States, Europe, and Asia over the subsequent 40 years [15]. Recently, EV71 has become an emerging life-threatening pathogen, particularly in the Asia-Pacific region [21,22]. EV71 possesses extensive tissue tropisms, involving the central nervous system and muscle, skeletal muscle, intestine, and immune cells, and can cause such fatal diseases as aseptic meningitis, paralysis, pulmonary edema, encephalomyelitis or even neurologic and psychiatric symptoms [21,22,23,24,25,26].

MicroRNAs (miRNAs) are a newly discovered class of small non-protein-coding RNAs that act via endogenous RNA interference [27,28,29,30,31,32]. To date, more than 1000 cellular miRNAs have been identified that mediate the post-transcriptional regulation of more than 30% of animal genes [28,33]. MiRNA biogenesis is initiated by RNA polymerase II or III, and the newly synthesized primary miRNA transcripts (pri-miRNAs) are cleaved by RNase III and Drosha into precursor miRNAs (pre-miRNAs). Precursor miRNAs are transported into the cytoplasm and further processed by Dicer to form miRNA-miRNA duplexes [34,35,36,37,38]. The miRNA-miRNA duplexes harbor a guide strand that generally functions as the mature miRNA. Mature miRNAs associated with an Ago protein form an RNA-induced silencing complex (RISC) by which miRNAs suppress specific mRNAs by targeting complementary sites of specific mRNAs mainly located in mRNA 3′ untranslated regions (3′ UTRs) [39,40,41]. Occasionally, 5′ UTRs and protein-coding regions can act as the potential binding sites for miRNAs [42,43,44,45]. By post-transcriptional regulation, miRNAs govern a wide range of biological functions, including cell proliferation, differentiation, apoptosis and host-pathogen interactions [46,47,48]. Certain DNA viruses encode viral miRNAs that are able to regulate viral or cellular gene expressions and to contribute to viral pathogenesis [49,50,51]. In contrast, some cellular miRNAs play a role in the virus life cycle, including viral genome replication and virus propagation [52,53].

In general, viruses are predisposed to evolve more genetic diversity to overcome environmental stresses and increase competitive advantages. However, this unique virus tendency generates various antigenic variations and potentiates drug resistance that impedes the development of effective antiviral therapies. Moreover, some viruses produce viral regulatory molecules, such as miRNAs and long non-coding RNAs (lncRNAs), to play a part in viral pathogenesis and life cycle [54,55,56,57]. On the other hand, viruses utilize cellular factors to hijack host bio-energy, evade immune attacks and enforce viral replication [58,59]. In this review, we discuss the emerging roles of cellular and virus-encoded miRNAs in host-pathogen interactions and provide potential strategies for antiviral therapies by manipulating such regulatory molecules.

## 2. Host Cellular miRNAs in Enterovirus Pathogenesis

### 2.1. Host miRNAs Participate in Antiviral Responses and Immune Escape in Enterovirus Infections

Generally, certain microbe-unique molecules, such as double-stranded RNA and cytidine-phosphate-guanosine DNA (CpG DNA), are recognized by host pattern-recognition receptors and activate endosomal toll-like receptor (TLR) signaling to produce type I interferons (IFNs), the first-line immune responses against viral infection [60,61,62,63]. The resulting IFNs can establish antiviral machinery in virus-infected subjects by inducing downstream interferon-stimulated genes, promoting T cell proliferation, stimulating IFNγ production and activating dendritic cells or natural killer cells [64,65,66,67]. Intriguingly, EV71 infection fails to elicit type I IFN production efficiently [68,69]. Ho and his colleagues found that miR-146a is induced in EV71 infection and further suppressed two critical components in interferon production, Interleukin-1 receptor-associated kinase 1 (IRAK1) and TNF receptor-associated factor 6 (TRAF6). Knocking out miR-146a or neutralizing virus-induced miR-146a restores IRAK1 and TRAF6 expression, augments IFNβ production, inhibits viral propagation and finally improves the survival of virus-infected neonatal mice [58]. In this case, the authors provided a clue to help develop preventive and therapeutic strategies against Enterovirus infections by manipulating miRNA expressions. Interferon regulatory factors can mediate the upregulation of miR-526a, which further targets Cylindromatosis (turban tumor syndrome), also known as CYLD, a well-known deubiquitinase, to enforce K63-linked RIG-I ubiquitination and then triggers IRF3 and NF-κB signaling. EV71 3C protease cleaves host Interferon regulatory factor 7 (IRF7) to block IRF-mediated miR-526a upregulation and then suppresses RIG-I-dependent IFNs production [70]. In addition to EV71, Coxsackie virus B3 (CVB3) infection also influences the host miRNA expression profile [71]. Zhang and his coworkers identified five differentially expressed miRNAs in CVB3-infected mice hearts using a miRNA microarray. As determined by bioinformatics analysis, these differentially expressed miRNAs might be involved in certain important immune and antiviral pathways, such as the Toll-like receptor signaling pathway, RIG-I-like receptor signaling pathway, NOD-like receptor signaling pathway, cytokine-cytokine receptor interaction, Mitogen-activated protein kinase (MAPK) signaling pathway, Janus kinase-Signal Transducer and Activator of Transcription (JAK-STAT) signaling pathway, and natural killer cell-mediated cytotoxicity. In contrast, the host also manipulates miRNA expression to establish immune attacks or eliminate viral pathogenesis [72,73]. CVB3-infected subjects activate immune responses to attack the virus, while elevated immune responses might result in cardiac myocyte destruction, reparative fibrosis, myocarditis and even heart failure [72,74]. Both miR-155 and miR-148a were upregulated in cardiac biopsies from viral myocarditis patients caused by CVB3 infection. RelA, an important molecule in NF-κB signaling, was demonstrated *in vitro* as the direct target of these two upregulated miRNAs [72]. Subsequently, the regulatory role of miR-155 was demonstrated in mouse cardiac myocytes and in a mouse infection model due to the sequence conservation of miR-155 between human and mouse. MiR-155 targets RelA and functions as a negative regulator in the host immune system, by which cardiac myoblast cytokine expression is reduced and the infected subjects are protected from an overdriven immune response; thus, the survival is improved in CVB3 infection [72]. In addition, miRNA-548 mimics present an inhibitory effect on IFNλ1 by targeting its 3′ UTR to regulate the IFNλ1-mediated antiviral response [73]. Li *et al.* [73] highlighted miR-548 as downregulated in both Vesicular stomatitis virus and EV71 infections, whereas hosts might establish an antiviral response. In these cases, hosts and viruses regulate cellular miRNAs expression to establish immune attacks against the virus infections or evolve immune escape machineries to create a beneficial situation for viral replication, respectively.

### 2.2. Host miRNAs Are Involved in Enterovirus Infection-Induced Apoptosis

**Figure 1:**
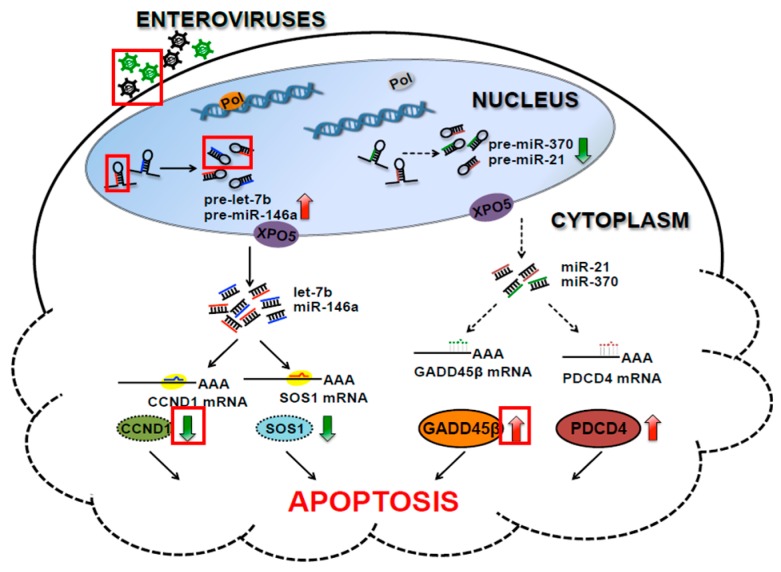
Cellular miRNAs are involved in Enterovirus infection-induced apoptosis. Hexagons indicate Enterovirus, hairpins indicate pre-miRNAs, red arrows indicate upregulated expression and green arrows indicate downregulated expression.

Apoptosis is an important cellular defense mechanism, particularly in the early phase of pathogen infection, by which pathogen-infected cells can be eliminated. Although viral spread can be partly restricted, apoptosis may cause tissue damage in the late stages of infection. Several studies have indicated that apoptosis is the major pathogenic feature of Enterovirus infection, resulting in host cell death and tissue damage [75,76,77]. Hence, the roles of miRNAs in Enterovirus infection-induced apoptosis were investigated [55,78,79]. The researchers found that miR-21 expression was suppressed in CVB3-induced myocarditis, while ectopic miR-21 expression significantly alleviated CVB3-induced myocarditis, including myocardial injury recovery, myocarditis score reduction and increased survival rate [78]. Programmed Cell Death 4 (PDCD4) was validated as the other important miR-21 target involved in apoptosis in several cell types [80,81,82]. The authors illustrated the underlying molecular mechanism in which miR-21 serves as an anti-apoptotic molecule in viral myocarditis via targeting PDCD4 [78]. On the other hand, EV71 infection induced let-7b expression in SH-SY5Y cells, which directly targets cyclin D1 (CCND1), a key element in cell cycle and apoptosis processes [79]. In contrast, the inhibition of let-7b by 2′-*O*-Methyl-RNA restored CCND1 expression, reduced the G2/M phase and restored SH-SY5Y proliferation. This evidence suggests that the proliferation retardation and apoptosis of EV71-infected cells are at least partly attributed to miRNA-mediated mechanisms [79]. More recently, Chang and her colleagues found that miR-146a is upregulated, while miR-370 is downregulated in EV71 infection [55]. Son Of Sevenless Homolog 1 (SOS1) and Growth arrest and DNA-damage-inducible β (GADD45β) are the targets of miR-146a and miR-370, respectively. Further functional studies indicated that the silencing of miR-146a restores SOS1 expression and partially attenuates EV71-induced apoptosis, while the ectopic expression of miR-370 decreases EV71-induced GADD45β expression and diminishes apoptosis. Moreover, the co-expression of antagomiR-146a and miR-370 showed an additive effect on attenuating EV71-induced apoptosis [55]. These studies clearly demonstrate that host miRNAs play a critical role in Enterovirus infection-induced apoptosis by regulating the key elements that are involved in cellular apoptosis and imply that miRNAs might act as potential therapeutic candidates by attenuating Enterovirus infection-induced apoptosis [55,78,79]. The miRNAs that are involved in the regulation of Enterovirus-induced apoptosis are elucidated in Figure 1 and summarized in Table 1.

Apoptosis is the major pathogenic feature of Enterovirus infections. MiR-21, which targets Programmed Cell Death 4 (PDCD4), is suppressed in CVB3-induced myocarditis and serves as an anti-apoptotic molecule. EV71-induced let-7b directly targets cyclin D1 (CCND1) and contributes to the retardation of host cell proliferation and the trigger of apoptosis. The other two altered miRNAs that were identified in EV71 infection, miR-146a and miR-370, target SOS1 and GADD45β, respectively. The suppression of SOS1 by miR-146a and the relief of GADD45β by miR-370 induce the apoptosis of EV71-infected host cells. These cellular miRNAs play critical roles in Enterovirus infection-induced apoptosis by regulating the key elements in the cellular apoptosis process.

### 2.3. Enterovirus Regulates Host Signaling Modulators Resulting in Pathogenesis

Many studies have clearly indicated that Enteroviral proteases cleave host factors and then lead to cellular or tissue malfunctions, such as apoptosis, translation shutdown and cytokine suppression, and finally cause pathogenesis [83,84,85,86,87,88,89,90]. For example, EV68 3C protease cleaved host cell TIR-domain-containing adapter-inducing interferon-β (TRIF), a key molecule downstream of TLR3, to abolish NF-κB signaling and IFN-β production and enabled viruses to escape the host innate immune response [83]. In addition to viral proteases, miRNAs have also been characterized as a critical players in host signaling transduction [54,55,58,78,79,91,92,93,94,95,96,97]. In the investigation of Coxsackie virus-induced cardiovascular pathogenesis, the destruction of cell-cell interactions is one of its key mechanisms. Several components of membrane structure are the targets of Coxsackie virus-regulated miRNAs. CBV3-induced miR-21 enhances desmin degradation by targeting a deubiquitinating enzyme, YOD1 [54], and Gap junction protein alpha 1 (GPJ1) is the target of CBV3-induced miR-1 [93]. MiR-21 also activated mitogen-activated protein kinase (MAPK) signaling cascade by targeting sprouty homolog 1 (SPRY1), a native inhibitor of fibroblast growth factor (FGF) pathways [94]. Moreover, CBV3 infection triggered ERK1/2 activation, leading to miR-126 upregulation. The miR-126 played as a pivot to link the ERK1/2 and WNT/β-catenin signaling pathways and promoted CBV3 propagation [96].

**Table 1:** MicroRNAs(miRNAs) regulations in Enteroviruses infections.

miRNA	Target	Enterovirus	Expression	Process	Model	Reference
miR-146a	IRAK1	EV71	Upregulation	Immune response	*In vitro* & *In vivo*	[58]
miR-146a	TRAF6	EV71	Upregulation	Immune response	*In vitro* & *In vivo*	[58]
miR-526a	CYLD	EV71	Downregulation	Immune response	*In vitro*	[70]
miR-155	RelA	CVB3 & VMC ^a^	Upregulation	Immune response	*In vitro* & *In vivo*	[72]
miR-148a	RelA	CVB3 & VMC	Upregulation	Immune response	*In vitro*	[72]
miR-548	IFNλ1	EV71 & HBV-infected subjects ^b^	Downregulation	Immune response	*In vitro*	[73]
miR-21	PDCD4	CVB3	Downregulation	Apoptosis	*In vitro* & *In vivo*	[78]
let-7b	CCND1	EV71	Upregulation	Cell cycle and Proliferation	*In vitro*	[79]
miR-146a	SOS1	EV71	Upregulation	Apoptosis	*In vitro*	[55]
miR-370	GADD45b	EV71	Downregulation	Apoptosis	*In vitro*	[55]
miR-21	YOD1	CVB3	Upregulation	Cell-cell interaction	*In vitro*	[54]
miR-1	GPJ1	CVB3	Upregulation	Cell-cell interaction	*In vitro*	[93]
miR-21	SPRY1	VMC & DCM ^c^	Ectopic	MAPK signaling	*In vitro*	[94]
miR-126	LRP	CVB3	Upregulation	Wnt/β-catenin signaling	*In vitro*	[96]
miR-126	WRCH1	CVB3	Upregulation	Wnt/β-catenin signaling	*In vitro*	[96]
miR-1246	DLG3	EV71	Upregulation	Cell death signaling	*In vitro*	[95]
miR-27a	EGFR	EV71	Downregulation	EGFR signaling	*In vitro*	[97]
miR-141	eIF4E	EV71	Upregulation	Protein synthesis	*In vitro*	[59]
miR-296-5p	EV71 VP1	EV71	Upregulation	Viral replication	*In vitro*	[56]
miR-296-5p	EV71 VP3	EV71	Upregulation	Viral replication	*In vitro*	[56]
miR-23b	EV71 VP1	EV71	Downregulation	Viral replication	*In vitro*	[98]
miR-342-5p	CVB3 2C	NA ^d^	Ectopic	Viral replication	*In vitro*	[99]
miR-373	EV71 5′ UTR	NA	Ectopic	Viral replication	*In vitro*	[100]
miR-542-5p	EV71 5′ UTR	NA	Ectopic	Viral replication	*In vitro*	[100]
miR-10a*	CVB3 3D	NA	Ectopic	Viral replication	*In vitro*	[57]

^a^ determined in CVB3 infection and viral myocarditis subjects (VMC); ^b^ determined in EV71 infection and HBV-infected subjects; ^c^ determined in viral myocarditis subjects (VMC) and dilated cardiomyopathy subjects (DCM); ^d^ not determined in virus infection.

Next, we focused on the role of miRNAs in EV71-induced neurological pathogenesis due to the severe neuronal complications in EV71-infected patients [21,24]. The EV71-induced miR-1246 directly repressed the expression of disc-large homolog 3 (DLG3), which is a member of the membrane-associated guanylate kinase protein family and is associated with mental disorders [95]. MiR-1246 might contribute to EV71-associated neurological pathogenesis by targeting DLG3. In addition to the direct destruction of neuronal components, the inflammatory reaction is one of main causes contributing to tissue damage, such as meningitis and encephalitis [101,102]. The increased Cyclooxygenase-2 (COX-2) and prostaglandin E2 (PGE2) induced by Enterovirus infection accelerated EV71 replication and reactive oxygen species (ROS) generation, and the upstream regulation mechanisms have been intensively elucidated [103,104,105]. Among these regulatory mediators, EGFR signaling plays a crucial role in EV71 replication in the human neuroblastoma cell line, SK-N-SH cells [103,104]. Zhang and his colleagues found that the expression of miR-27a is downregulated during EV71 infection and that miR-27a could target EGFR [97]. Further studies have shown that the ectopic expression of miR-27a suppresses EGFR expression and reduces Akt and ERK phosphorylation. Finally, the blockage of the EGFR signaling cascade attenuated EV71 replication [97]. The miRNAs that are involved in the regulation of host signaling pathways are summarized in Table 1.

### 2.4. Host miRNAs Are Involved in Enterovirus Infection-Induced Protein Synthesis Shutdown

It has long been believed that the viral protease-mediated cleavage of host factors that are involved in the host cap-dependent translation process might almost or even thoroughly account for the shutdown of host protein synthesis caused by Picornavirus infection [85,86,90,106,107]. Enterovirus 2A and 3C proteases digested the host eukaryotic initiation factor 4G (eIF4G) and Poly(A)-binding protein (PABP), and these cleavages led to the shutdown of host cell protein synthesis and further promoted apoptosis, along with nuclear condensation and DNA fragmentation [85,86,90]. In addition to viral proteases, miRNAs have also been characterized as critical players in host protein synthesis shutdown and signaling regulation [54,55,59,78,79,91,92,93,94,95,96,97,108]. The role of miRNAs in the virus-induced translational switch has recently begun to be investigated. Ho and his colleagues found that cellular miR-141 is induced by EV71 infection, which targets eIF4E, the cap-dependent translation initiation factor, and results in the shutdown of host protein synthesis, while the silencing of virus infection-induced miR-141 almost recovers host protein synthesis and blocks viral propagation up to 1000-fold [59]. Hence, virus infection-induced miR-141 expedites the translational switch from cap-dependent translation to cap-independent translation. This study largely increased the understanding of how miRNA facilitates Enterovirus-causing host protein synthesis in addition to the classical concept [59,109].

## 3. Host miRNAs Are Involved in the Enterovirus Life Cycle

### 3.1. Cellular miRNAs Target Vial Genome to Suppress Viral Replication

**Figure 2:**
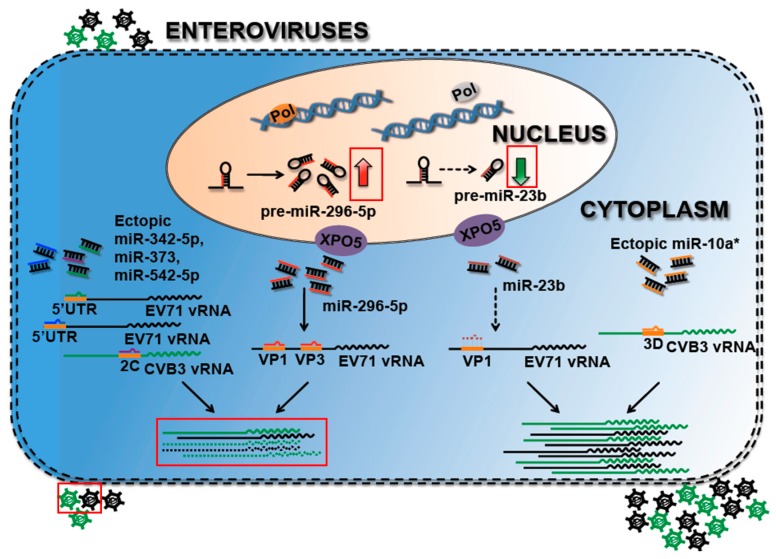
Host miRNAs are involved in the Enterovirus life cycle by targeting to viral genomes. Hexagons indicate Enterovirus, solid lines indicate viral RNAs, dotted lines indicate inhibited viral RNAs, red arrows indicate upregulated expression and green arrows indicate downregulated expression.

The host and virus influence cellular miRNA expressions during virus infection, and the altered miRNAs target cellular molecules to enforce viral pathogenesis or establish host defense machineries. Recently, several studies have suggested that host miRNAs could target viral genomes and result in the suppression of virus replication [56,57,98,99,100]. The induction of miRNA-296-5p was found in EV71-infected human rhabdomyosarcoma (RD) and SK-N-SH cells, and the silicon analysis results predicted that miRNA-296-5p might target both the VP1 and VP3 regions of the viral genome; this speculation was confirmed by molecular biological assays (Figure 2) [56]. Meanwhile, the suppression of endogenous miR-296-5p promoted EV71 replication, and the introduction of mutations into binding sites on the viral genome led EV71 to escape the inhibitory effects of miR-296-5p [56]. Wang *et al.* [99] and Wen *et al.* [98] and also found similar phenomena in CVB3 and EV71 infections, respectively [98,99]. The expression of miR-23b was downregulated in EV71 infection, while the restoration of miR-23b by specific mimic inhibited EV71 production (Figure 2). A further study has evidenced that miR-23b suppressed EV71 replication by targeting the EV71 VPl protein [98]. Wang and the coworkers discovered that miR-342-5p could significantly inhibit CVB3 replication by directly targeting the 2C-coding region of the viral genome (Figure 2) [99]. Both miR-373 and miR-542-5p target the 5′ untranslated region (5′ UTR) of EV71 vRNA and thus attenuate viral propagation as assayed in RD cells (Figure 2) [100].

Cellular miRNAs play a regulatory role in the viral life cycle to suppress or promote virus replication by targeting the viral genome. MiR-296-5p is upregulated, while miR-23b is downregulated in EV71 infection. EV71 infection-induced miR-296-5p could target both viral VP1 and VP3 regions and result in the inhibition of virus replication. The downregulation of miR-23b in EV71 infection relieves EV71 viral RNAs (vRNAs) from miRNA-mediated inhibition. In addition to endogenous miRNAs, the ectopic introduction of miRNAs also affects virus replication. The ectopic expression of miR-342-5p, miR-373 or miR-542-5p suppresses Enterovirus replication, but miR-10* facilitates CVB3 replication. MiR-342-5p targets the CVB3 2C-coding region and thus inhibits CVB3 replication. In contrast, miR-10a* acts as a functional RNA molecule to facilitate CVB3 replication by targeting the viral 3D-coding region. Similarly, miR-373 and miR-542-5p attenuate EV71 propagation by targeting the 5′ UTR of EV71 vRNAs.

### 3.2. Cellular miRNAs Target the Viral Genome to Promote Viral Propagation

Intriguingly, miR-10a*, the passenger strand of the miRNA duplex, is a functional RNA molecule in CVB3 infection, in which miR-10a* directly targets the nt6818-nt6941 sequence of the CVB3 3D-coding region, facilitating CVB3 replication (Figure 2) [57]. Furthermore, miR-10a* was abundant in the cardiac tissues of suckling Balb/c mice, indicating that miR-10a* might be involved in CVB3-induced myocarditis [57]. Despite a large number of reports evidently showing that miRNAs suppress target gene expression via the RISC complex, a few cases have shown that miRNAs are capable of positively regulating target gene expressions [53,110,111]. As mentioned in 2005, a host miRNA, miR-122, targeted Hepatitis C virus (HCV) 5′ noncoding region, facilitating viral RNA replication by promoting RNA folding or sequestration in the replication complexes [53]. This evidence implied that miRNAs might serve as potential therapeutic agents for Enterovirus infection treatment. The miRNAs that are involved in the manipulation of viral genomes are provided in Figure 2 and summarized in Table 1.

## 4. Conclusions and Perspective

A complete molecular understanding of virus pathogenesis is necessary to develop more efficient strategies against virus infection. Since the discovery of miRNAs in 1993, miRNA is becoming the largest family of gene regulators, accounting for the regulation of one-third of human genes. In the case of Enterovirus infection, virus-induced cellular miRNAs modulate the cellular and infection processes and contribute to pathogenesis by targeting either host mRNAs or virus RNAs. This growing evidence demonstrates the important role of miRNAs in Enterovirus infection-related pathogenesis and accelerates the progress of anti-virus strategies. There are no effective antiviral treatments, approved medications or vaccines for Enterovirus infections except for Poliovirus [16,112,113]. Routine treatment is supportive for reducing clinical symptoms. The efficacy of passive immunoglobulin treatments is not often effective or satisfying for infected subjects. Unfortunately, quarantine might be the best solution to prevent Enterovirus outbreaks. Although anti-EV vaccines are undergoing clinical trials and may soon be approved, the cross-protection of these vaccines requires further evaluation. Herein, miRNAs provide an opportunity to develop alternative miRNA-based therapeutic and preventive strategies for non-polio Enterovirus infections. In fact, the phase I clinical trial of MRX34, a miR-34 mimic, in solid tumors and hematological malignancies is expected to be completed by the end of 2015 [114]. A phase IIa clinical trial was conducted to assess the safety and efficacy of Miravirsen, a 15-nucleotide locked nucleic acid-modified antisense oligonucleotide against mature miR-122 in HCV carriers, and the preliminary results showed exciting therapeutic potential with acceptable adverse drug effects (ADE) [115]. Furthermore, the phase II clinical trial of RG101, another anti-miR-122 antagonist, combined with an anti-HCV agent in HCV patients has been underway since August 2015. For EV infection control, miR-146a-based medication provides new insight into the potential anti-EV71 therapy by showing that the neutralization of EV71-induced miR-146a prevents death, as demonstrated in an EV71 infection mouse model via the restoration of type I interferon production [58]. The potential long-term cytotoxicity and genotoxicity might not be a critical consideration in EV infections compared to chronic infections or cancers due to the short therapeutic period. Moreover, targeting host components is a new strategy to overcome the drug resistance and antigenic variation of viruses, particularly RNA viruses. Since the mystery of miRNAs in Enterovirus infection is generally understood, the preventive and therapeutic activities of miRNAs might be conspicuous in the foreseablefuture.
